# Functional Dicer Is Necessary for Appropriate Specification of Radial Glia during Early Development of Mouse Telencephalon

**DOI:** 10.1371/journal.pone.0023013

**Published:** 2011-08-03

**Authors:** Tomasz Jan Nowakowski, Karolina Sandra Mysiak, Thomas Pratt, David Jonathan Price

**Affiliations:** Developmental Biology Laboratory, Centre for Integrative Physiology, University of Edinburgh, Edinburgh, United Kingdom; VIB & Katholieke Universiteit Leuven, Belgium

## Abstract

Early telencephalic development involves transformation of neuroepithelial stem cells into radial glia, which are themselves neuronal progenitors, around the time when the tissue begins to generate postmitotic neurons. To achieve this transformation, radial precursors express a specific combination of proteins. We investigate the hypothesis that micro RNAs regulate the ability of the early telencephalic progenitors to establish radial glia. We ablate functional Dicer, which is required for the generation of mature micro RNAs, by conditionally mutating the *Dicer1* gene in the early embryonic telencephalon and analyse the molecular specification of radial glia as well as their progeny, namely postmitotic neurons and basal progenitors. Conditional mutation of *Dicer1* from the telencephalon at around embryonic day 8 does not prevent morphological development of radial glia, but their expression of Nestin, Sox9, and ErbB2 is abnormally low. The population of basal progenitors, which are generated by the radial glia, is disorganised and expanded in *Dicer1^-/-^* dorsal telencephalon. While the proportion of cells expressing markers of postmitotic neurons is unchanged, their laminar organisation in the telencephalic wall is disrupted suggesting a defect in radial glial guided migration. We found that the laminar disruption could not be accounted for by a reduction of the population of Cajal Retzius neurons. Together, our data suggest novel roles for micro RNAs during early development of progenitor cells in the embryonic telencephalon.

## Introduction

The embryonic forebrain (prosencephalon) comprises the telencephalon, which generates the cerebral cortex and the basal ganglia, and the diencephalon, which generates the prethalamus and the thalamus. Just after closure of the neural tube, the telencephalon is a thin neuroepithelium surrounding the ventricles. Proliferation of neuroepithelial stem cells adjacent to the ventricles leads to the thickening of the telencephalon. Between embryonic day 9.5 (E9.5) and E10.5 in mouse, the neuroepithelial stem cells mature and elaborate their radial processes to become radial glia [1], which are the progenitor cells during subsequent neurogenesis. Radial glial cells generate neurons and intermediate (basal) progenitors (reviewed by [2]); the latter divide away from the ventricular surface to generate neurons. Newly generated neurons migrate towards the pial surface along the processes of radial glia to form the postmitotic cell layer [3], [4], [5], [6].

Mouse Dicer is a type III endoribonuclease encoded by the *Dicer1* gene [7], which catalyzes the cleavage of double stranded RNA molecules [8]. Mature micro RNAs (miRNAs) are 21-27nt products of Dicer activity [9], [10]. They interact with complementary sequences on protein coding messenger RNA molecules (mRNAs), mainly in the 3′ untranslated regions [11], [12]. This interaction is recognised and sustained in the RNA-induced silencing complex (RISC) [13], [14], [15] and regulates expression via transcript degradation by endoribonucleolytic cleavage, deadenylation and decapping, or translational inhibition [16], [17]. This process takes place in the processing (P-) bodies [18], which require RNA for assembly and can maintain mRNA in an untranslated state [19].

Mice null for *Dicer1* are not viable past E7.5 [20], indicating that the endogenous RNA interference pathway is critical for mammalian development. To bypass early embryonic lethality and investigate the role of miRNAs in telencephalic development, three mouse mutant lines carrying conditional deletions of *Dicer1* in the forebrain have been thoroughly analysed. In these lines, expression of cre-recombinase is driven by *Emx1*, *Nes* or *Camk2* promoters [21], [22], [23]. The overall conclusion from these studies is that the cells primarily affected by loss of Dicer are postmitotic neurons. Migration of neuronal precursors to the postmitotic layers as well as their subsequent survival are compromised in the mutant tissue, whereas apical and basal progenitor cell populations are not affected detectably until later, after E14.5, when abnormally large proportions of them undergo apoptosis [22], [23].

A number of studies have shown that miRNAs are involved in mouse embryonic stem cell proliferation and differentiation [24], [25], [26]. It remains possible that Dicer is required in the early telencephalic progenitor population in vivo and that previous experiments did not address this. One possibility is that Dicer is required by this population earlier than previously addressed; another is that it might be required in aspects of their biology, such as cell identity, that have not been examined before. To generate a very early deletion of *Dicer1* restricted mainly to the telencephalon we used a *Foxg1^cre^* allele. Foxg1 is expressed by all cells in the telencephalic anlage at the anterior end of the neural tube from before neural plate closure. *Foxg1^cre^* expresses cre in the telencephalic progenitors from around E8.0 [27]. We found that *Foxg1^cre^*-induced ablation of *Dicer1* results in abnormal protein expression by neural progenitor cells (radial glia) at E11.5 coinciding with the generation of the first postmitotic neurons. Both basal progenitors and postmitotic neurons, which are normally produced by the radial glia at E11.5, are misplaced through the depth of the telencephalic wall, yet their proportional contribution to the total number of cells in the tissue is not reduced.

## Results

### Effects of *Dicer1*-mutation on telencephalic miRNA levels

**Figure 1:**
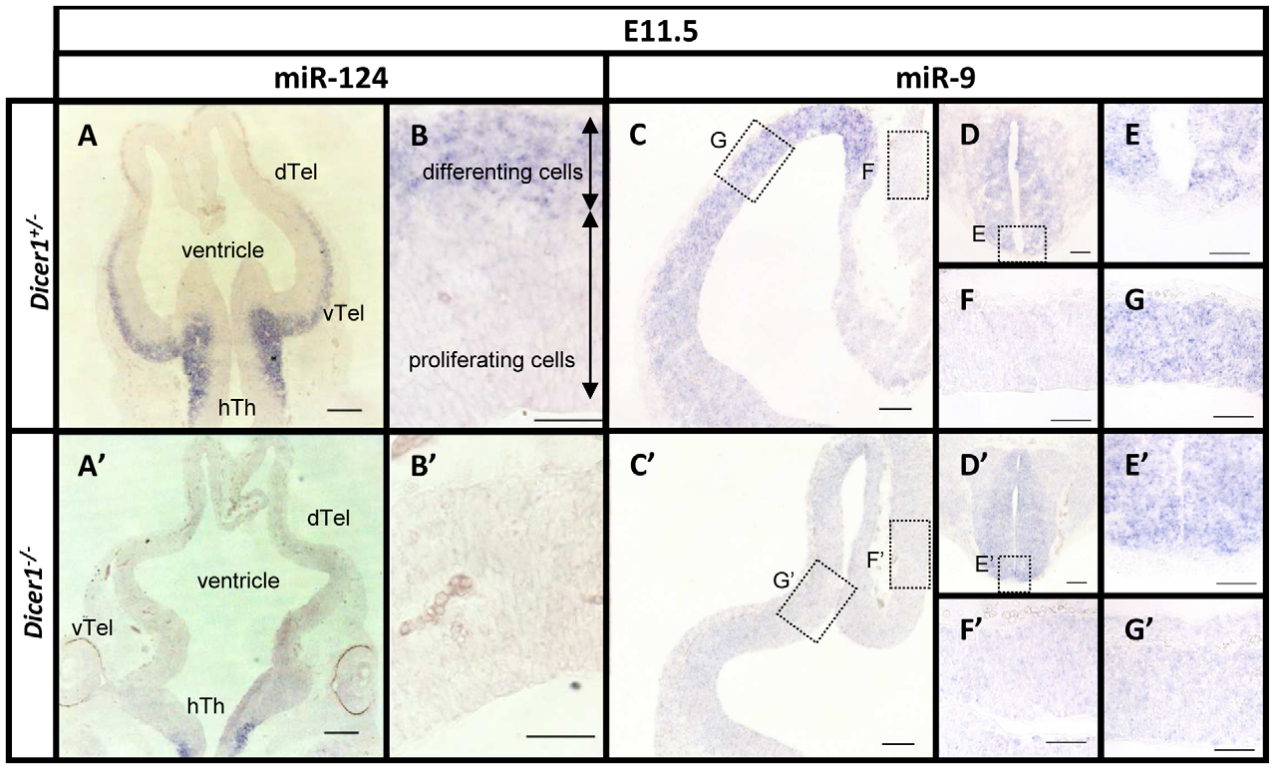
Loss of mature miR-124 and miR-9 in the telencephalon of *Dicer1^-/-^* embryos. Two abundant brain miRNAs, miR-124 and miR-9 were detected using LNA *in situ* hybridisation. At E11.5, mature miR-124 was expressed in the postmitotic layers (A, B). Mature miR-124 was not detected in *Dicer1^-/-^* telencephalon but its expression was maintained in hypothalamus (A', B'). In control embryos at E11.5, miR-9 was strongly expressed throughout the thickness of the dorsal telencephalon (C) and the spinal cord (D). High power images of the staining in the spinal cord (E), the diencephalon, which was devoid of mature miR-9 (F) and the dorsal telencephalon (G) in *Dicer1^-/-^* embryos. Mature miR-9 was depleted from the dorsal telencephalon by E11.5 (C', G'). Expression of mature miR-9 in the spinal cord of the *Dicer1^-/-^* embryos was not altered (D', E'). High magnification image of the diencephalon (F') is included as a negative control. Abbreviations: dTel: dorsal telencephalon, vTel: ventral telencephalon, hTh: hypothalamus. Scale bar: (A, A') - 200 µm, (B, B', E, E', F, F', G, G') - 50 µm, (C, C', D, D') - 100 µm, .

To confirm the anticipated effects of *Dicer1* deletion on mature miRNA production, we examined the expression of the two most abundant miRNAs in the E11.5 brain [28], [29]: miR-124, whose expression is restricted to the post-mitotic neuronal population [22], [30] and miR-9, which is expressed in both the progenitor and postmitotic cells [31]. Whereas in the forebrain of control *Dicer1^+/-^* embryos we detect mature miR-124 in the post-mitotic cell layer (Figure 1 A, B, C), in *Dicer1^-/-^*embryos miR-124 expression is absent from the telencephalon and retained only in postmitotic cells in the hypothalamus, which expresses low levels of *Foxg1* (Figure 1 A', B').

In the forebrain of control embryos, mature miR-9 is present throughout the thickness of the dorsal telencephalic wall (Figure 1 C, G) and in the spinal cord at E11.5 (Figure 1 D - E) but was undetectable in the diencephalon (Figure 1 C, F). In the dorsal telencephalon of embryos with *Foxg1*-driven *Dicer1* deletion we found the level of mature miR-9 was depleted by E11.5 and was undetectable both in the cortex and in the diencephalon (Figure 1 C', F', G'). Mature miR-9 expression in the spinal cord, where Foxg1 is not normally expressed, was unaffected in the *Dicer1^-/-^* embryos (Figure 1 D', E').

These results indicate that *Dicer1^-/-^* embryos show a telencephalon-specific depletion of miRNAs by E11.5.

### Dicer-deficient neuroepithelial stem cells maintain their cell identity

**Figure 2:**
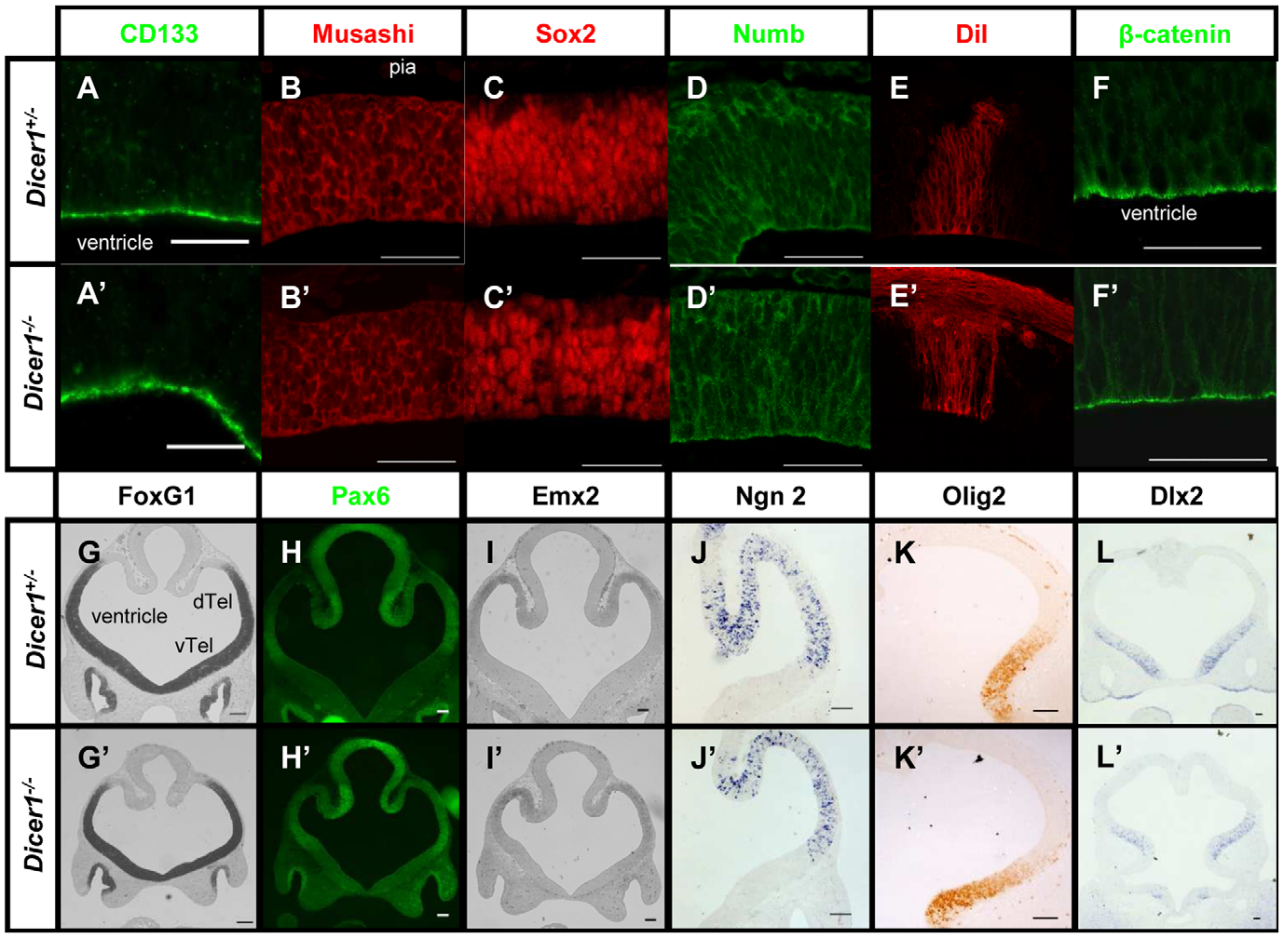
Expression of markers of neuroepithelial stem cells is normal in *Dicer1^-/-^* telencephalon. In panels A–E and A'–E' tissue is oriented with ventricular surface at the bottom and pia at the top of the image. CD133/Prominin1 (A), Musashi (B), Sox2 (C) and Numb (D) are expressed by the proliferative neuroepithelial stem cells of embryonic telencephalon. Expression of these genes is maintained in *Dicer1^-/-^* dorsal telencephalon (A' – D' respectively). Radial processes were labelled using the lipophilic dye DiI to show gross morphology of the radial glia in control (E) and *Dicer1^-/-^* telencephalon (E') showing no change in the latter. Enriched β-catenin immunostaining at the ventricular surface of the control telencephalon (F) was also unaffected by the loss of functional Dicer (F'). A normal pattern of *Foxg1* expression was obtained using RNA in situ hybridisation in control telencephalon (G) and *Dicer1^-/-^* telencephalon (G'). Pax6 (H), *Emx2* (I), *Ngn2* (J), Olig 2 (K) and *Dlx2* (L) are involved in dorso-ventral patterning of the telencephalon and the expression of these markers was not affected by the loss of Dicer (H' – L' respectively). Abbreviations: Di: Diencephalon, dTel: dorsal telencephalon, vTel: ventral telencephalon. Scale bar: (A) – (F') - 50 µm, (G) – (L') - 100 µm.

To analyse the molecular identities of early telencephalic progenitors, we examined the expression of markers of neuroepithelial stem cells as well as the expression of markers of radial glia. We immunostained coronal E11.5 telencephalon sections to detect the presence of four proteins normally expressed in the most undifferentiated neuroepithelial progenitors: stem cell marker CD133 (Prominin1); Notch signalling inhibitor Numb; Numb inhibitor Musashi; and Sox2 (sex determining region Y box 2) [32], [33], [34], [35], [36]. The vast majority of cells in the E11.5 control telencephalon are immunoreactive for CD133, Musashi, Sox2, and Numb. In *Dicer1*-/- telencephalon, expression of CD133, Musashi, Sox2 and Numb appeared similar to that in controls (Figure 2 A–D, A'–D'). To assess the mitotic activity of the progenitor cells we quantified the average proportion of phosphorylated histone-3 (pHH_3_) immunoreactive cells directly lining the ventricular surface at E11.5. In control embryos, an average of 25.7±6.7 (sem) % of cells in the dorsal telencephalon and 25.6±5.5 % of cells in the ventral telencephalon were pHH_3_ immunopositive. In *Dicer1*-/- embryos these values were not significantly different with 28.3±2.8 % of cells in the dorsal telencephalon (p>0.05, n = 3) and 26.2±2.1 % of cells in the ventral telencephalon (p>0.05, n = 3) immunopositive for pHH_3_ (Figure S1). Furthermore, expression of genes involved in forebrain patterning was investigated and we found no alterations in the dorso-ventral extent of the expression domains of the following transcription factors at E11.5: the pan-telencephalic marker FoxG1 (Figure 2 G, G') [37], dorsal telencephalic markers Pax6 (Figure 2 H, H') [38], Emx2 (Figure 2 I, I') [39] and Ngn2 (Figure 2 J, J') [4] or ventral telencephalic markers Olig2 (Figure 2 K, K') [40], [41] and Dlx2 (Figure 2 L, L') [40]. Overall, these results indicate that many aspects of neuroepithelial stem cell identity are not affected by the loss of Dicer.

### Radial progenitors are defective in their expression of Nestin

Around E10, the neuroepithelial stem cells generate the neurogenic progenitors, radial glia. This transformation includes elaboration of the radial processes that run between the pial and ventricular surfaces of the telencephalic wall [1]. We labelled the radial processes using DiI placed on the pial surface at E11.5 and found no difference between radial processes in *Dicer1^-/-^* and in control (*Dicer1*+/−) tissue (Figure 2 E, E'). Previous experiments in which the radial processes of the telencephalic progenitor cells were disrupted showed alterations in the expression pattern of proteins that play a role in cell adhesion at the apical surface of the telencephalon, such as β-catenin [42]. We performed immunostaining for β-catenin and found no difference between *Dicer1^-/-^* and control tissue (Figure 2 F, F').

**Figure 3:**
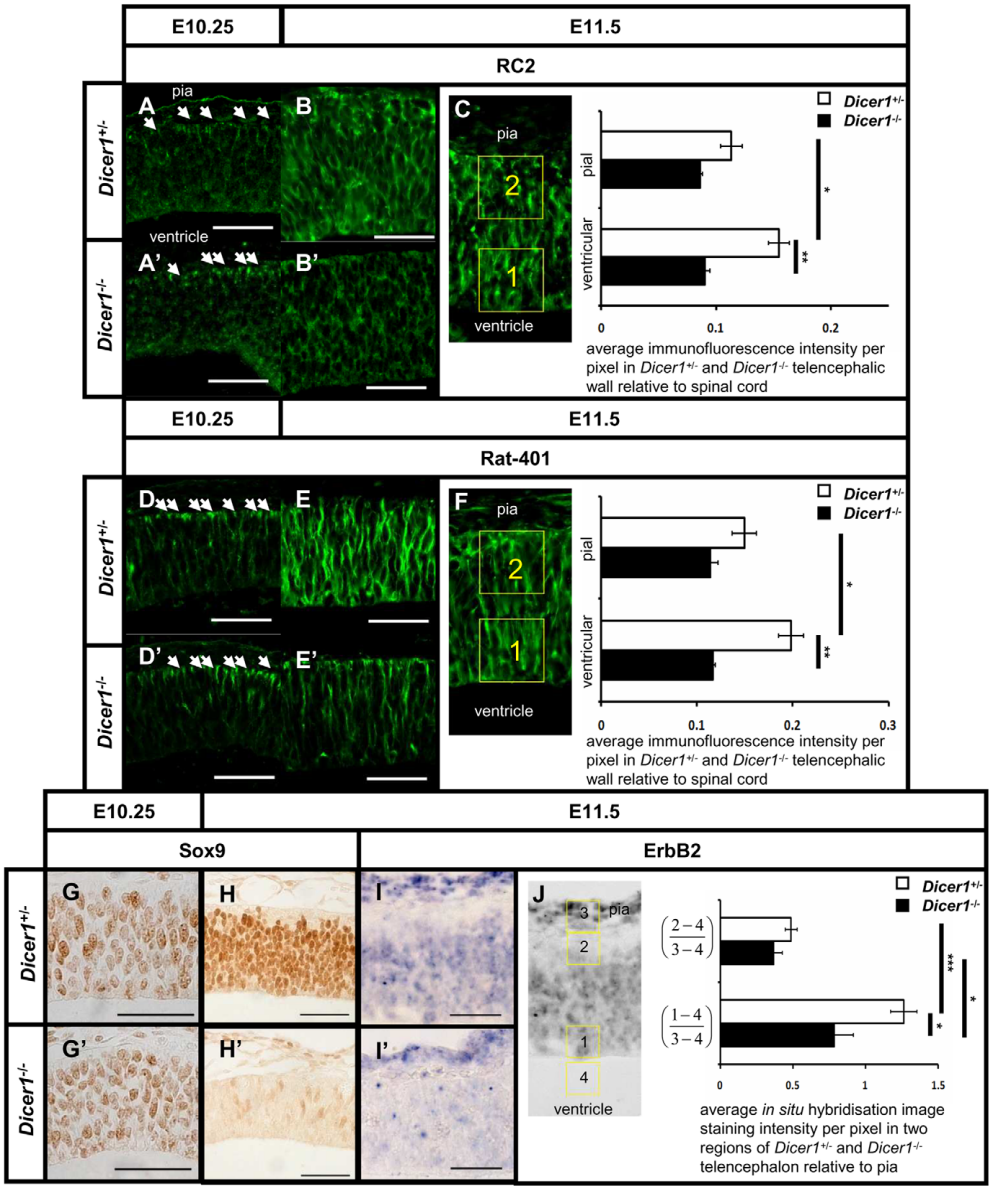
Expression of Nestin, Sox9 and ErbB2 is compromised in Dicer deficient telencephalon. The RC2 antigen is encoded by the *Nes* gene and is expressed in the basal end-feet of the radial processes of the neuroepithelial progenitor cells at E10.25 (A). In mutant tissue this pattern of expression was maintained (A'). Around the onset of neurogenesis when neuroepithelial stem cells give rise to radial glia, RC2 expression becomes elaborated by E11.5 (B). This expansion was compromised in the *Dicer1^-/-^* telencephalon (B'). Quantification of immunofluorescence intensity for RC2 antigen in ventricular and pial regions of the control (open bars) and *Dicer1^-/-^* telencephalon (filled bars) revealed significant reduction of immunostaining in the ventricular region. Similarly to RC2, Rat-401 is also encoded by the *Nes* gene and its expression follows the pattern of RC2 expression in the end feet of radial processes at E10.25 (D) and also becomes expanded by E11.5 (E). In the *Dicer1^-/-^* telencephalon, Rat-401 expression is present in the end feet of the radial processes (D') but is reduced compared to control at E11.5 (E'). Quantification of immunofluorescence intensity revealed a significant reduction in the *Dicer1^-/-^* telencephalon in the ventricular region (F). Sox9 transcription factor expression is present in the vast majority of proliferative cells in the dorsal telencephalon at E10.25 (G) and E11.5 (H). In *Dicer1^-/-^* dorsal telencephalon only a few cells are immunoreactive for Sox9 (H'). ErbB2 is a receptor for Neuregulin1 and is expressed strongly in proliferative cells in control telencephalon as well as the pia (I). In *Dicer1^-/-^* telencephalon the ErbB2 mRNA in situ hybridisation staining was strong in the pia, but faint in the proliferative cells (I'). Expression levels of ErbB2 mRNA were quantified as described in Results, standardising the levels of staining both in pial and ventricular regions to the level of staining in the pia to show a significant reduction of staining in the ventricular region (J). Scale bar: 50 µm, *p<0.05, **p<0.01, ***p<0.001, error bars indicate s.e.m.

Nestin is a type IV intermediate filament protein expressed in neural progenitor cells. Two commonly used antibodies against Nestin, the Rat-401 and RC2 clones, recognise its two different isoforms whose expression is largely but not entirely identical [43], [44]. Staining using the RC2 antibody was used as the defining criterion of radial glial identity [1]. Progenitors at the neuroepithelial stem cell stage (E10.25) express Nestin mainly at the pial end-feet of the radial processes both in control (Figure 3 A, D, arrows) and *Dicer1^-/-^* telencephalon (Figure 3 A', D'). By E11.5 the expression of Nestin is strongly enhanced throughout the radial processes of the progenitor cells (Figure 3 B, E). In the *Dicer1^-/-^* telencephalon, expression of both isoforms is lower compared to control levels (Figure 3 B', E').

To quantify the abnormality at E11.5, sections from 4 control and 3 *Dicer1^-/-^* embryos were immunofluorescently labelled simultaneously using identical conditions with Rat-401 or RC2 antibodies. Fluorescence image intensity was recorded from 50 µm x 50 µm areas (‘boxes’) placed at 300 µm separation along the dorso-ventral extent of the telencephalon. The boxes were positioned adjacent to the ventricular edge (Figure 3 C, F, box ‘1’) as well as adjacent to the pial membrane (Figure 3 C, F, box ‘2’). Intensity recorded from an identical box that was placed on an image of the spinal cord from the same section (150 µm ventral to the roof plate, adjacent to the central canal) was used for normalisation. For each control and *Dicer1^-/-^* section analysed, average intensities per pixel from the series of boxes ‘1’ and boxes ‘2’ along the dorso-ventral extent of the telencephalon were normalised to those from the spinal cord in the same section since, as described above, the spinal cord was unaffected by the mutation (this corrects for any residual variation in staining for technical reasons between different sections). Both RC2 and Rat-401 immunostaining intensities were significantly lower in the *Dicer1^-/-^* than in the control telencephalon; in the pial region the differences were not significant (Figure 3 C, F). In controls, immunofluorescence intensities for both Nestin isofoms became significantly higher in the ventricular region than in the pial region (Figure 3 C,F), which was a reversal of the pattern in both control and *Dicer^-/-^* telencephalon at E10.25; this did not occur in *Dicer^-/-^* telencephalon. Taken together, our results indicate that loss of Dicer from the telencephalon inhibits the normal enrichment of Nestin protein in the ventricular region by E11.5.

To test if this difference could be caused by different overall cell density in *Dicer1^-/-^* tissue compared to control, densities of DAPI-stained cells in dorsal and ventral telencephalic regions were quantified. We found that in control embryos average densities were 22±4*103 cells/mm2 in the dorsal telencephalon and 29±6*103 cells/mm2 in the ventral telencephalon. These values were not significantly different in *Dicer1^-/-^* embryos, which had 20±5*103 cells/mm2 in the dorsal telencephalon (p>0.05, n = 3) and 24±6*103 cells/mm2 in the ventral telencephalon (p>0.05, n = 3).

### Dicer deficient progenitors lose expression of transcription factor Sox9 and the ErbB2 receptor

Previous studies using non-neural tissues identified the transcription factor Sox9 as a co-enhancer for the expression of the *Nes* gene [45]. Immunohistochemical staining of the telencephalon around E10.25 and E11.5 reveals that while in control tissue virtually all progenitor cells are stained using the antibody against Sox9 transcription factor at both ages (Figure 3 G, H), in *Dicer1^-/-^* dorsal telencephalon the expression is present at E10.25 (Figure 3 G') and almost completely absent at E11.5 (Figure 3 H').

The ErbB2 receptor has been implicated in radial glia formation since it mediates Neuregulin1 signalling that leads to RC2 expression [46]. *In situ* hybridisation for ErbB2 mRNA in control dorsal and ventral telencephalon shows ErbB2 is normally expressed throughout the progenitor layer and in the pia overlying the neuroepithelium (Figure 3 I). In *Dicer1^-/-^* tissue the ErbB2 mRNA is present in the pia but strongly reduced in the progenitor layer (Figure 3 I'). To quantify this observation for control (n = 4) and *Dicer1^-/-^* (n = 3) sections, we used a densitometric method as described above with minor modifications. A series of 30 µm x 30 µm boxes were placed over the telencephalic tissue adjacent to the ventricular edge (Figure 3 J, box ‘1’), adjacent to the pial edge in the differentiating preplate (Figure 3 J, box ‘2’) and over the pia for normalisation (Figure 3 J, box ‘3’). A reading from a box placed over the ventricle was used to measure background (Figure 3 J, box ‘4’) which was subtracted from values in the boxes above it. Values from box ‘1’ (ventricular region) and box ‘2’ (preplate region) were normalised against the corresponding value for the pia (box ‘3’). In both control tissue and *Dicer1^-/-^* tissue, average intensity in the ventricular region is significantly higher than that in the preplate region. The average intensity in the ventricular region is, however, significantly lower in the *Dicer1^-/-^* than in control telencephalon (Figure 3 J). This indicates that ErbB2 expression is significantly reduced in the ventricular zone in *Dicer1^-/-^* telencephalon.

We also examined expression of some of these markers at a later age. At E12.5 in control telencephalon, the expression pattern of Sox9 largely overlaps that of Sox2 in the progenitor cell layer (Figure S2 A–F). In *Dicer1^-/-^* tissue the size of the Sox2 expression domain is markedly reduced, yet the remaining cells retain intense immunofluorescent labelling (Figure S2 A'–C') while the expression of Sox9 is largely absent (Figure S2 D'–F'). The size of the TuJ1-marked population of postmitotic cells is also greatly reduced in the E12.5 *Dicer1^-/-^* telencephalon (Figure S2 A'-C') compared to control telencephalon (Figure S2 A–C). The radial glial fibre expression of Nestin (Rat-401) is also greatly reduced in the E12.5 *Dicer1^-/-^* telencephalon (Figure S2 D'–F') in comparison with abundant labelling in control tissue (Figure S2 D–F). These data are consistent with our observations from E11.5 embryos, showing a selective reduction in the expression of radial glial markers, including Sox9 and Rat-401. Together, these findings indicate that the loss of functional Dicer causes persistent incorrect specification of radial glia.

### Loss of functional Dicer results in the expansion of the basal progenitor population and misplacement of postmitotic neurons

Radial glia are progenitor cells and during normal development begin to generate postmitotic neurons as well as intermediate (basal) progenitor cells around E11.5. They also guide the radial migration of neuronal progeny to generate cortical layers [6], [47] and disruption of radial glia results in mislocalised neurons [48]. Previous studies identified defects in the capacity of Dicer deficient stem cells to differentiate [24], [25]. We anticipated that disruption of radial glia at E11.5 in *Dicer1^-/-^* telencephalon could cause defects in the capacity of these progenitors to generate postmitotic neurons and/or guide migration normally.

**Figure 4:**
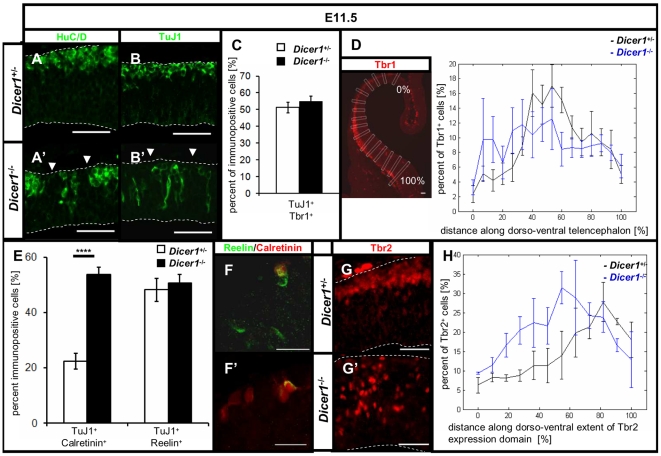
Neurons and basal progenitors generated by radial precursors are affected by the loss of Dicer. HuC/D is normally expressed in early postmitotic neurons and immunostaining for HuC/D is enriched in the postmitotic layer forming coherent staining beneath the pia (A). In *Dicer1^-/-^* telencephalon HuC/D expressing cells are misplaced through the thickness of the telencephalic wall and the staining in the layer beneath the pia is discontinuous with numerous gaps lacking staining beneath the pial membrane (A', arrows). Similarly, TuJ1 is expressed by early postmitotic neurons (B). In Dicer deficient telencephalon, TuJ1 immunoreactive cells are scattered through the depth of the neuroepithelium (B'). Tbr1 is expressed by differentiated neurons at E11.5 and labels about half of the TuJ1-positive cells in control and *Dicer1^-/-^* telencephalon (C). Quantification of the distribution of Tbr1-immunopositive cells as a proportion of total number of cells along the dorso-ventral extent of its expression domain revealed that differentiation of postmitotic neurons was not affected anywhere in the telencephalon by E11.5 following the loss of Dicer (D). Calretinin and Reelin are markers of Cajal-Retzius cells, which are involved in regulating the radial migration of postmitotic neurons to the postmitotic layer. Quantification revealed a significant increase in the proportion of TuJ1 cells that were Calretinin double-positive in *Dicer1^-/-^* telencephalon compared to control whilst the proportion of TuJ1 cells that were Reelin double-positive was unaltered by the loss of functional Dicer (E). Expression of Reelin and Calretinin is not overlapping in all cells (F, F'). Tbr2 is expressed by the basal progenitors in control telencephalon (G). In *Dicer1^-/-^* telencephalon, Tbr2 immunoreactive cells are misplaced through the depth of the telencephalic wall (G') and their proportion was increased compared to control tissue (H). Dashed lines outline the ventricular and pial edges of the telencephalic wall. Scale bar: 50 µm. *p<0.05, ****p<10^−5^ error bars indicate s.e.m.

To address this hypothesis we looked at the pattern of expression of markers of early-born neurons, mRNA binding protein HuC/D and type III beta tubulin (TuJ1). In control telencephalon at E11.5, cells immunoreactive for HuC/D (Figure 4 A) or TuJ1 (Figure 4 B) were located beneath the pial membrane forming a coherent layer. In *Dicer1*-/- tissue the TuJ1 and HuC/D immunostaining revealed a pattern that was discontinuous with numerous gaps of staining beneath the pial membrane (Figure 4 A', B', arrows). In addition, a number of neurons were misplaced through the depth of the telencephalic wall. The overall proportions of TuJ1+ cells in the *Dicer1*-/- telencephalon (15.6±1.7%) and the control telencephalon (19.1±1.5%), irrespective of their location, were not significantly altered (Student's t-test, p>0.05, embryos n = 9). These findings indicate that early telencephalic progenitors generate correct proportions of neurons after Dicer deletion, but many of those neurons migrate abnormally.

To test if loss of functional Dicer compromises the ability of early postmitotic neurons to complete differentiation, we quantified the proportion of TuJ1 neurons that also express a marker of more mature postmitotic neurons, Tbr1 [49]. We found that about half of the TuJ1 immunopositive cells express Tbr1 both in control and *Dicer1^-/-^* telencephalon (Figure 4 C). In addition, we counted the proportions of cells (visualised with DAPI) that were Tbr1 positive along the dorso-ventral extent of the telencephalon (for counting strategy see Materials and Methods as well as Figure 4 D). We found no difference between control and *Dicer1^-/-^* telencephalon (Figure 4 D, two way ANOVA, p>0.05, n = 9).

One possible explanation for misplacement of postmitotic neurons could be a loss of Cajal Retzius cells [50]. During forebrain development the Cajal-Retzius neurons in the telencephalon have been shown to originate from progenitors located in the ventricular zone of the telencephalon as well as other regions of the brain ([51] and references therein). The misplacement of neurons in *Dicer1^-/-^* telencephalon might result from an absence of Cajal-Retzius cells. To test this, we quantified the proportion of TuJ1-positive cells that were double-positive for either Reelin or Calretinin, both of which mark the Cajal-Retzius neurons at E11.5 [52], [53]. We found no difference between genotypes in the proportion of TuJ1 positive cells that expressed Reelin; the proportion of TuJ1 positive cells that co-expressed Calretinin was actually significantly increased in *Dicer1^-/-^* telencephalon compared to control (Figure 4 E, p<10–5, n = 9). We conclude that there is no evidence to suggest that misplaced neurons could be accounted for by a depletion of Cajal-Retzius cells from *Dicer1^-/-^* telencephalon.

Using double immunohistochemistry for Reelin and Calretinin (Figure 4 F, F') we found that the proportion of Reelin positive neurons that co-expressed Calretinin were not significantly different in *Dicer1^-/-^* telencephalon (58.6±5.3%) and control tissue (53±4.2%) (Student's t-test p>0.05, n = 9). This indicates that the subpopulation of TuJ1 neurons that is expanded in *Dicer1^-/-^* tissue expresses Calretinin but not Reelin.

Beside neurons, radial glia generate basal progenitors. During early telencephalic development, around E11.5 – E12.5, the transcription factor Tbr2 (Eomesodermin) is expressed by the basal progenitors and a small subset of Tbr1 positive postmitotic neurons in the dorsal telencephalon [54] (Figure 4 G). In *Dicer1^-/-^* tissue, Tbr2 positive cells were scattered through the depth of telencephalic wall (Figure 4 G'), reflecting the migration defect described above. We quantified the proportion of telencephalic cells expressing Tbr2 along the dorso-ventral extent of its expression domain (see Materials and Methods) and found that the proportion was increased above control levels in all but the most ventral part of the *Dicer1^-/-^* dorsal telencephalon (Figure 4 H, two way ANOVA, p<0.01, n = 3).

### The proportion of apoptotic cells is increased at E11.5

**Figure 5:**
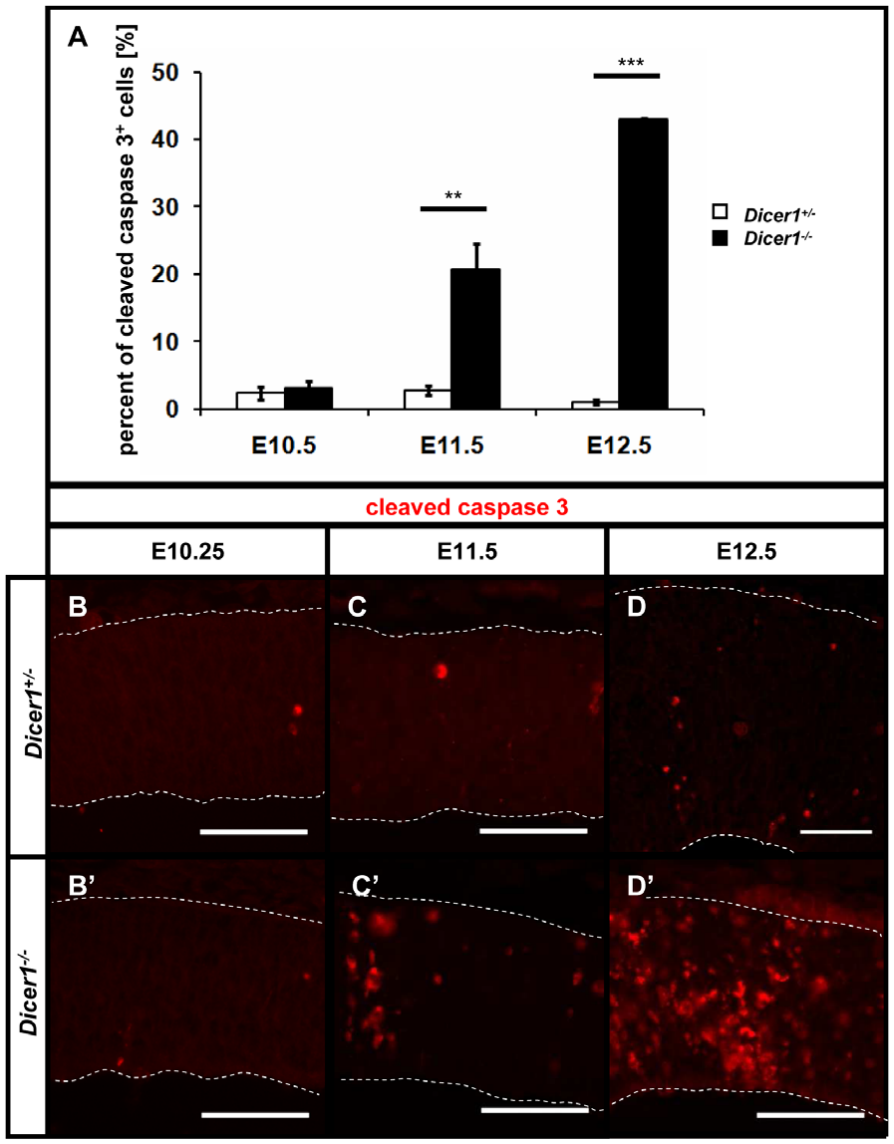
E11.5 is the first embryonic day when apoptosis becomes significant. (A) Percent of cells in the telencephalon of control (open bars) and *Dicer1^-/-^* telencephalon (filled bars) was quantified at E10.25, E11.5 and E12.5 showing that E11.5 is the first stage at which there is a significantly higher number of cleaved caspase-3 immunopositive cells due to the loss of functional Dicer. Immunohistochemical staining of control tissue reveals only a small proportion of cells stain for cleaved caspase-3 in either E10.25 (B), E11.5 (C) or E12.5 (D) control telencephalon, as well as E10.25 *Dicer1^-/-^* telencephalon (B'). E11.5 is the first stage when significant levels of apoptosis are observed (C') and they are further exacerbated by E12.5 (D'). Scale bar: 50 µm, **p<0.01, ***p<0.001 error bars indicate s.e.m.

Several studies have indicated that cell survival is compromised following the loss of Dicer in the telencephalon [21], [22], [23], [55]. We immunostained control and *Dicer1^-/-^* telencephalon at E10.25 and at E11.5 with an antibody against cleaved caspase 3 and quantified the proportion of apoptotic cells in the telencephalon (Figure 5 A). While the proportion of cleaved caspase 3 positive cells is not significantly different between genotypes (n = 3) at E10.25 (Figure 5 B, B'), at E11.5 it is significantly increased (p<0.01, n = 3) in *Dicer1^-/-^* telencephalon compared to control tissue with immunoreactive cells scattered throughout the depth of the telencephalic wall (Figure 5 C, C').

We then investigated whether apoptosis could have influenced tissue development by E11.5. In control embryos, the average thickness of the telencephalic wall is 90±5 (sem) µm dorsally and 145±15 µm ventrally. These values are not significantly different in the *Dicer1^-/-^* embryos where the average thicknesses of the dorsal and ventral telencephalic wall are 80±5 µm (p>0.05, n = 3) and 130±20 µm (p>0.05, n = 3). As described above, cell density is not different between genotypes. These data indicate that E11.5 is the first stage when apoptosis becomes significant and that prior to E11.5 cell death has not significantly affected the development of the telencephalon.

By E12.5, the proportion of apoptotic cells increased in the *Dicer1^-/-^* telencephalon (Figure 5 A, D'). The average thicknesses of the dorsal and ventral telencephalic wall in *Dicer1^-/-^* embryos were 85±4 µm and 136±12 µm respectively, significantly lower than in control tissue, which had corresponding values of 170±8 µm (p<0.001, n = 3) and 286±32 µm (p<0.01, n = 3). By E14.5, the size of the telencephalon was greatly reduced (Figure S3 B) compared to controls (Figure S3 A), presumably resulting from escalating cell death after E12.5.

## Discussion

Here we describe an analysis of early telencephalic development in mature miRNA deficiency using *Foxg1^cre^Dicer1^fl/fl^* mice. We analysed the expression of markers of neuroepithelial stem cells as well as more restricted neural progenitor cells (radial glia) and found strong evidence that around the time of development when radial glia (as defined in [1]) are normally generated, expression of Sox9, ErbB2 and Nestin proteins is compromised. We also found that in the absence of functional Dicer protein, radial progenitors generate normal proportions of postmitotic neurons and an increased proportion of basal progenitor cells. Both of these cell types are misplaced through the depth of the telencephalic wall in *Dicer1^-/-^* telencephalon.

In previous work investigating which processes during early telencephalic development depend on miRNA maturation three conditional mouse mutant models of *Dicer1* gene mutation were studied. First, the *Emx1^cre^* line leads to early recombination in the dorsal telencephalon and results in a reduction of the postmitotic cell layer through apoptosis. The newborn neurons that are generated fail to migrate to the post-mitotic layer. In this line, apical and basal progenitor cell populations are unaffected by the loss of mature miRNAs until well after the onset of apoptosis [22]. In the second model using *Nes^cre^,* cre-recombinase is expressed later than in the *Emx1^cre^* model. The deletion results in deficits in the number and migration of postmitotic neurons while the proliferative properties of the progenitor population are completely unaffected by the loss of miRNAs. Cell survival is not affected until perinatal stages of development when a significant number of apoptotic cells is observed in the ventricular and subventricular zones [23]. The third model utilizing *Camk2^cre^*, expresses active cre from E15.5, and the primary defect is postnatal apoptotic cell death. Anterior commissure pathfinding defects and a reduction in dendritic branching in the hippocampus have also been reported [21].

Our data using a cre strain that deletes earlier than in previous models indicate that in the absence of functional Dicer protein the neuroepithelial stem cells show defects in generating radial glial progenitors around the onset of neurogenesis. Neural progenitor cell identity is an aspect of neural development that has not to our knowledge been addressed in previous models of *Dicer1^-/-^* dorsal telencephalon. We found that the expression of radial glia markers RC2 and Rat-401 is severely diminished at E11.5, soon after the tissue begins to generate the first postmitotic neurons. Both antigens are encoded by the *Nes* gene. Their expression is present mainly in the basal end-feet of neuroepithelial stem cells and normally extends more widely when these generate radial glia [1], [3], [6]. This does not occur normally after loss of Dicer. Multiple pathways have been suggested to promote Nestin expression including Neuregulin-1 signalling through the ErbB2 receptor to promote RC2 expression in the telencephalon [46]. Null mutation of Nestin results in embryonic lethality, but no evidence has so far been found for alteration of cytoarchitecture and the exact role of Nestin remains to be unravelled. Here we propose that the level of Nestin expression is dependent upon the presence of mature miRNA processing enzyme Dicer. The effect of loss of miRNAs on Nestin might be secondary consequence of the loss of ErbB2 and/or Sox9. Importantly, Nestin is a commonly used marker for neuroepithelial malignancies [56] and further understanding of mechanisms regulating its expression may provide therapeutic targets in neuroepithelial tumour cells.

The expression of Sox9 protein is reduced between E10.25 and E11.5. According to recent reports, except for a few cases, most miRNAs control target mRNA stability by recruiting deadenylases such as CAF1 thereby reducing their stability [57], [58]. Therefore, loss of mature miRNAs is predicted to result in the stabilization of their direct targets and increased protein output. In the case of Sox9 whose mRNA is targeted by miR-124 during postnatal neurogenesis [59] we found that the Sox9 protein is lost throughout the dorsal telencephalic progenitors while miR-124 is lost from the postmitotic layer by E11.5 [30]. It is plausible that the loss of Sox9 reflects the altered radial progenitor identity and that the effect is indirect. Nonetheless, direct targeting of Sox9 mRNA by miRNAs other than miR-124, such as miR-9, remains a formal possibility as miRNAs have been shown to have the capacity to promote protein expression through non-canonical pathways, although very few examples have so far been reported [60], [61]. Thus, it is conceivable that loss of miRNAs might cause direct loss of Sox9 protein.

We found that the loss of Nestin coincides with the loss of Sox9 protein. In human melanoma cells Sox9 has also been identified as an important regulator of Nestin expression [45]. The reduction of Sox9 protein in the cortex is consistent with the reduction in the proportion of Sox9 positive cells in *Dicer1^-/-^* mutant retina [62], where Sox9 normally mediates the switch between neurogenesis and gliogenesis (reviewed in [63]) and promotes generation of the Müller glia [64]. In the telencephalon, Sox9 is known to promote the establishment and maintenance of neural stem cells and promote their potential to generate neurospheres [65]. Many signalling pathways have been implicated in the regulation of Sox9 expression in various tissues. Notably, these include molecules involved in forebrain development such as Fibroblast Growth Factor [66] Sonic hedgehog [65] and Notch1 [67]. Dicer protein catalyses the maturation of most mature miRNAs and it is possible that at least several key developmental pathways regulating telencephalic organogenesis would be disrupted. These signalling pathways have previously been shown to be disregulated in various Dicer deficient tissues as well as through a direct regulation by specific miRNAs [68], [69], [70], [71]. Additionally, Sox9 expression is also known to be regulated by pathways directly involved in cytoskeletal remodelling, such as RhoA [72], or Vimentin [73] and miRNAs have also been shown to play a role in regulating neuronal cytoarchitecture [74].

Apoptosis is the most widely reported phenotype of all *Dicer1^-/-^* tissues. Several mechanisms have been proposed including miR-24 regulation of caspase-3 [75] as well as regulation of PDCD4 or SOD1 in neurospheres [76]. It is possible that apoptosis is a secondary effect of Dicer loss. In *Nes^-/-^* mouse telencephalon a high proportion of cells undergoes apoptosis around E10.5 [77] making the loss of Nestin a novel candidate mechanism for the induction of apoptosis at early developmental stages of neural development. In addition, activated caspase-3 has been shown to cleave Dicer in a way that it gains an activity of a deoxyribonuclease, exacerbating cell death [78].

Given the misspecification of the radial glia, it is remarkable that the progenitors do not show an inability to generate the two classes of cells that they normally generate by E11.5: postmitotic Tbr1 positive neurons and the Tbr2 positive intermediate progenitor cells [2], [54], [79]. The increased proportion of cells expressing Tbr2 is in line with a previous prediction based on miRNA profiling of neural progenitor cells in rat dorsal telencephalon which proposed that the expression of miR-92 is down-regulated around the onset of neurogenesis and that it could be directly targeting Tbr2 for post-transcriptional repression [80].

The observation that the proportion of either TuJ1 or Tbr1 expressing cells is not altered in the *Dicer1^-/-^* telencephalon generated using the *Foxg1^cre^* allele seems to contrast with other studies looking at *in vivo* neurogenesis of cortical *Dicer1^-/-^* neuroepithelium [22], [23] as well as *Dicer1^-/-^* neural stem cells *in vitro* [70]. These studies established that the ability of Dicer-deficient progenitor cells to generate postmitotic neurons is compromised. It is possible that direct neurogenesis from the radial progenitors might also be compromised in our *Dicer1* mutation, in which the radial glial identity is incorrectly specified around the time when the progenitor cells begin to generate postmitotic neurons, but that the increased proportion of intermediate progenitors could compensate for this and, in turn, normal proportions of TuJ1- expressing cells could be generated in the mutant cortex by E11.5.

During normal corticogenesis, neurons generated prior to E11.5 contribute to the preplate. This is composed of several subpopulations including the Cajal-Retzius neurons, subplate and early projection neurons. Three commonly used markers of the very early-born neurons are Reelin, Tbr1 and Calretinin, with Reelin and Calretinin being expressed predominantly in Cajal-Retzius cells. The expression domains of these proteins are partially, but not completely, overlapping at E11.5 [81]. Disruption of the Cajal Retzius cell population has been shown to lead to disrupted organisation of the telencephalon [82]. While it was possible that the misplacement of neurons and basal progenitors following the loss of functional Dicer might have been caused by a loss of Cajal-Retzius cells, we found this was not the case. Failure to correctly specify radial glia cell identity is a possible explanation for the phenotype given that ablation of radial glia has been shown previously to cause defects in tissue organisation [48].

We found that the proportion of Calretinin-expressing but not Reelin-expressing TuJ1 cells is increased in *Dicer1^-/-^* telencephalon. It is interesting that the proportion of Tbr1 did not show a significant increase given that previous studies in the wild type have shown that all Cajal-Retzius cells express Tbr1 [81]. It is possible that this relationship does not hold in the *Dicer1^-/-^* telencephalon and that some Cajal-Retzius cells fail to express Tbr1. This would need further work to investigate the nature of Cajal-Retzius cells following the loss of functional Dicer.

**Figure 6:**
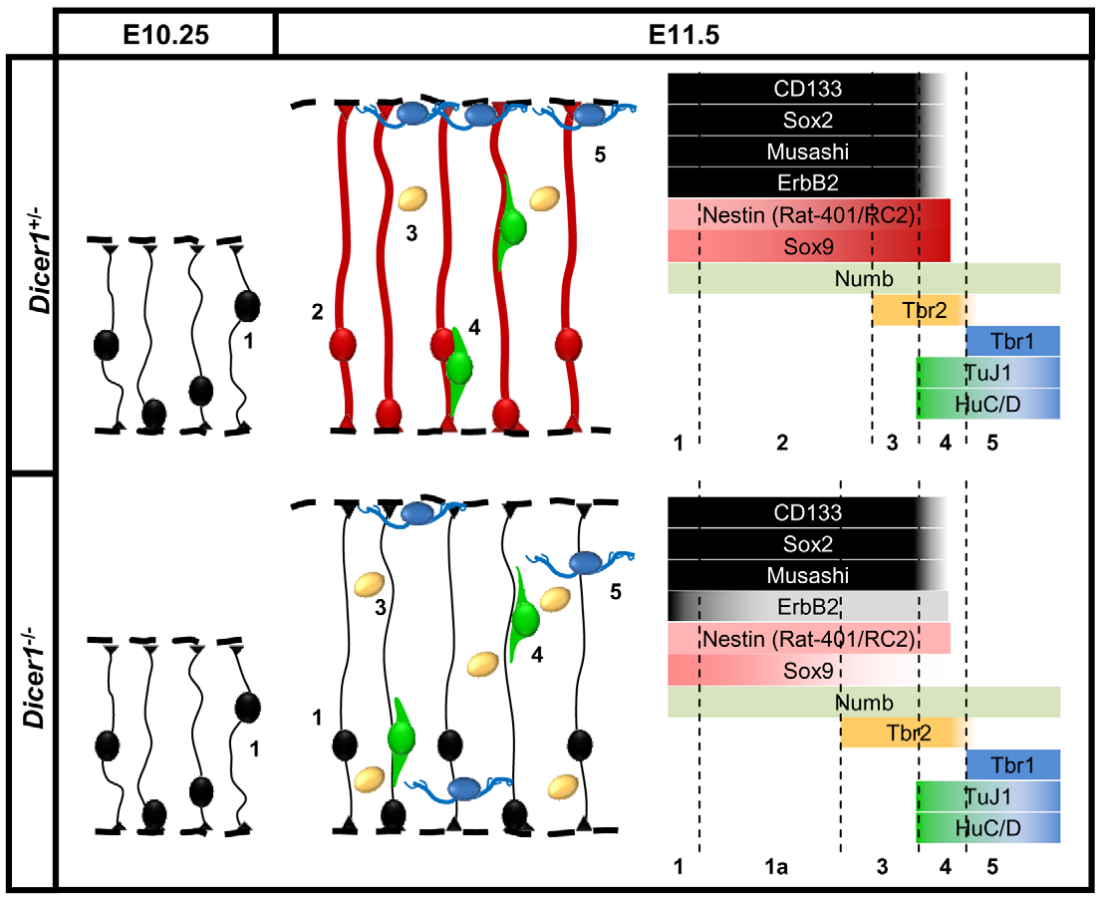
Changes to radial progenitors and their progeny in *Dicer1^-/-^* telencephalon. At E10.25 the telencephalon comprises the neuroepithelial stem cells “1” which express stem cell markers Sox2, Musashi, CD133. Expression of these proteins is maintained throughout their undifferentiated state. By E11.5, the neuroepithelial stem cells establish the radial glia “2”. Basal progenitors “3” and neurons “4, 5” are two classes of progeny generated by radial glia around E11.5. Dicer deficient neuroepithelium does not establish the appropriate molecular signature of radial glia at E11.5, which normally strongly express Nestin and Sox9 proteins. The proportion of Tbr2 positive basal progenitors is increased in *Dicer1^-/-^* telencephalon while the proportions of early postmitotic neurons labelled with TuJ1 and HuC/D or differentiated Tbr1 positive neurons are unchanged. The laminar organisation of both basal progenitors and neurons is disrupted following the loss of functional Dicer.

Taken together, our results provide a systematic description of the phenotype following the loss of functional Dicer protein early during telencephalic organogenesis. We propose that during the very early development of the neuroepithelium, the progenitor cells do not develop the appropriate molecular signature of the radial glia. We found that the basal progenitor population is expanded while the proportion of postmitotic neurons is not changed (Figure 6). Additionally, we found that postmitotic neurons are misplaced through the depth of the telencephalic wall and that the Cajal Retzius population, which is involved in regulating the appropriate migration of postmitotic neurons, is not reduced.

## Materials and Methods

### Animals

The licence authorising this work was approved by the University of Edinburgh's Ethical Review Committee on 22nd September 2008 (application number PL35-08) and by the Home Office on 6th November 2008. Animal husbandry was in accordance with the UK Animals (Scientific Procedures) Act 1986 regulations. *Foxg1^cre^* males [27] were first crossed to females homozygous for a transgenic allele of *Dicer1, Dicer1^fl/fl^*, which comprises a floxed exon 23 [68] encoding the majority of the RNase IIIb domain involved in the formation of the Dicer protein's active site [8]. Heterozygous *Foxg1^cre^Dicer^fl/+^* males were then used in timed mating experiments with *Dicer1^fl/fl^* females to generate *Foxg1^cre^Dicer1^fl/fl^* (*Dicer1^-/-^*) and control *Foxg1^cre^Dicer1^fl/+^* (*Dicer1^+/−^*) embryos at desired stages of development. Females were checked daily and the first day when vaginal plug was detected was designated as E0.5. To minimise animal suffering, pregnant dams were culled by cervical dislocation under terminal anaesthesia according to the Code of Practice for Humane Killing of Animals under Schedule 1 to the Animals (Scientific Procedures) Act 1986 issued by the Home Office. Genotyping was performed as described previously [68]. Embryonic age was determined based on the number of visible somites and other morphological features such as retina pigmentation, auditory hillocks and nasal pits. Embryos with 30–34 somites were designated as E10.25. Embryos with 45 – 47 somites were designated as E11.5 and embryos with well defined tongue, retina pigmentation but lacking 5 rows of whiskers were designated as E12.5. Harvested embryos were fixed in either Feketes fixative or 4% paraformaldehyde in 0.1 M phosphate buffer (pH = 9.0) either for 4 hr at room temperature or overnight at 4°C. Embryos were subsequently either processed into paraffin blocks and stored at room temperature or cryoprotected and frozen in either 1∶1 mixture of OCT (Fisher Scientific) and 30% sucrose in 0.1 M phosphate buffer (pH = 7.4) or 7.5% gelatine, 15% sucrose in 0.1 M phosphate buffer (pH = 7.4) and stored at −70°C.

### Immunohistochemistry

Sections were cut serially at either 6 µm (paraffin) or 14 µm (cryosections) and reacted according to standard protocols. Heat induced antigen retrieval was achieved by microwaving in 10 mM sodium citrate buffer, pH = 6. Primary antibodies used were against CD133 (1∶100, Millipore), Musashi (1∶10, Cell Signaling), β-catenin (1∶400, BD Biosciences), Reelin (1∶1000, Chemicon), β-tubulin type III (TuJ1, 1∶400, Sigma), Pax6 (1∶50, Developmental Studies Hybridoma Bank (DSHB), University of Iowa, Iowa City, IA), Rat-401 (1∶100, DSHB), RC2 (1∶100, DSHB), Numb (1∶100, Abcam), calretinin (1∶1000, Swant), Sox9 (1∶1500, Millipore), Sox2 (1∶100, Millipore), Olig2 (1∶500, DSHB, University of Iowa, Iowa City, IA), cleaved caspase-3 (Asp175) (1∶50, Cell Signaling), Tbr1, Tbr2 (1∶1000, Englund et al., 2005, gift from R.Hevner, University of Washington, Seattle, WA), HuC/HuD (1∶150, Invitrogen), phosphorylated histone 3 (ser10) (1∶100, Cell Signaling). Binding was revealed either using an appropriate Alexa-488 or Alexa-564 conjugated secondary antibodies (1∶200, Invitrogen), or using an appropriate biotinylated secondary antibody (1∶200, Dako) with the avidin– biotin- peroxidise system (Vector Laboratories). Where appropriate, the nuclear counterstain DAPI was applied (Vector Laboratories)

### *In situ* hybridisation

*In situ* hybridisation was performed as described before [83]. The hybridisation temperature for miRNA *in situ* hybridisations using locked nucleic acid probes (LNA) was determined as melting temperature −21°C (miRNA *in situ* hybridisation). The following RNA probes were used for *in situ* hybridisations: Dlx2 (generous gift from John Rubenstein), Emx2 (generous gift from Antonio Simeone), Erbb2 (generous gift from Carmen Birchmeier), Foxg1 [37], generous gift from Thomas Theil), mmu-miR-124-1 (Exiqon, DK), mmu-miR-9 (Exiqon, DK), Ngn2 (generous gift from Thomas Theil). All sections were mounted with Aquatex mounting medium (Merck).

### Quantification of immunopositive cells

To quantify the proportions of cleaved caspase-3 and phosphorylated histone 3 (pHH3) [84] immunopositive cells, 6 µm paraffin sections from 3 *Dicer1^-/-^* and 3 *Dicer1^+/-^* E11.5 embryos were immunostained as described above. One section from rostral and one from central telencephalon were analysed in each brain for each antigen, along with an additional DAPI counterstained section. For every section a series of images was taken covering the entire dorso-ventral extent of the telencephalon at 40x magnification for each antigen and for DAPI-counterstained cell nuclei. Counting boxes (100 µm wide, running through the entire depth of the telencephalic wall) were positioned along the dorso-ventral extent of the telencephalon at constant separation of 100 µm and were aligned with their base along the ventricular edge. Tissue thickness was estimated to the nearest 12.5 µm along the side of each counting box. For caspase-3, numbers of positive cells were counted through the depth of the telencephalic wall. To determine overall cell densities, counts of cell nuclei were averaged over three adjacent sections from either central or rostral telencephalon and divided by the average area of the counting boxes. For pHH3 immunopositive cells, we recorded the proportions of cells that were positive for pHH3 along the ventricular edge for each counting area.

To quantify the proportions of cells in *Dicer1^+/-^* and *Dicer1^-/-^* telencephalon that were double-positive for TuJ1 and either Tbr1, Reelin or Calretinin, or that were positive for Tbr2, a similar approach was taken to the quantification described above except that counting boxes of 50 µm width were placed along the dorso-ventral extent of the tissue at constant 75 µm separation (Figure 4D).

### DiI labelling

Use of the lipophilic dye DiI (Molecular Probes) has been described before [85]. We followed the procedure as described for labelling radial glia [41].

### Microscopy

Images of diaminobenzidine (DAB) immunostained tissue and *in situ* hybridisation sections were imaged using a Leica microscope connected to a Leica DFC480 digital camera. Immunofluorescent sections were imaged using a Leica microscope connected to a Leica DFC 360 FX digital camera. Confocal images of DiI labelled tissue were acquired using Zeiss LSM150 confocal system. Image intensity measurements were performed using Matlab R2009a (Mathworks).

### Statistical analysis

Student's t-tests were performed using Microsoft Office Excel (Microsoft) and two-way analysis of variance (two-way ANOVA) was performed using Matlab R2009a (Mathworks).

## Supporting Information

Figure S1 **Proportion of mitotic cells remains unaltered after the loss of functional Dicer.** Immunohistochemical staining using an antibody against phosphorylated (ser10) Histone 3 (pHH3) reveals that at E11.5 most immunoreactive cells are located directly at the ventricular surface (A) and loss of functional Dicer did not cause this pattern to be disrupted (A'). Quantification did not reveal any changes in the proportion of mitotic cells in either dorsal or ventral telencephalon (B). Scale bar: 50 µm, error bars indicate s.e.m.
(TIF)

Figure S2 **Dicer deficient telencephalon is severely disrupted by E12.5.** Immunohistochamical staining for TuJ1 marks the postmitotic neurons in the telencephalon (A, C). This population is greatly reduced in *Dicer1^-/-^* telencephalon (A', C'). Sox2 marks the proliferative population (B, C), which is also diminished in the *Dicer1^-/-^* tissue (Figure S2 B', C'). At E12.5, radial glia express Rat-401 (D, F) as well as Sox9 (E, F) and in the *Dicer1^-/-^* telencephalon only a small fraction of radial processes can be detected (D', F') and the expression of Sox9 is greatly reduced (E', F'). Scale bar: 100 µm.
(TIF)

Figure S3 **The volume of the** ***Dicer1^-/-^*** **telencephalic tissue is greatly reduced by E14.5.** Hematoxylin and eosin staining of coronal sections through the brain at E14.5 (A) reveals a hugely abnormal telencephalon in the *Dicer1^-/-^* telencephalon (B). Scale bar: 100 µm.
(TIF)
