# Supplementary figures and images for: Functional Dicer Is Necessary for Appropriate Specification of Radial Glia during Early Development of Mouse Telencephalon
Source: PLoS One. 2011 Aug 3;6(8):e23013. doi: 10.1371/journal.pone.0023013 (PMC3149632; doi:10.1371/journal.pone.0023013)

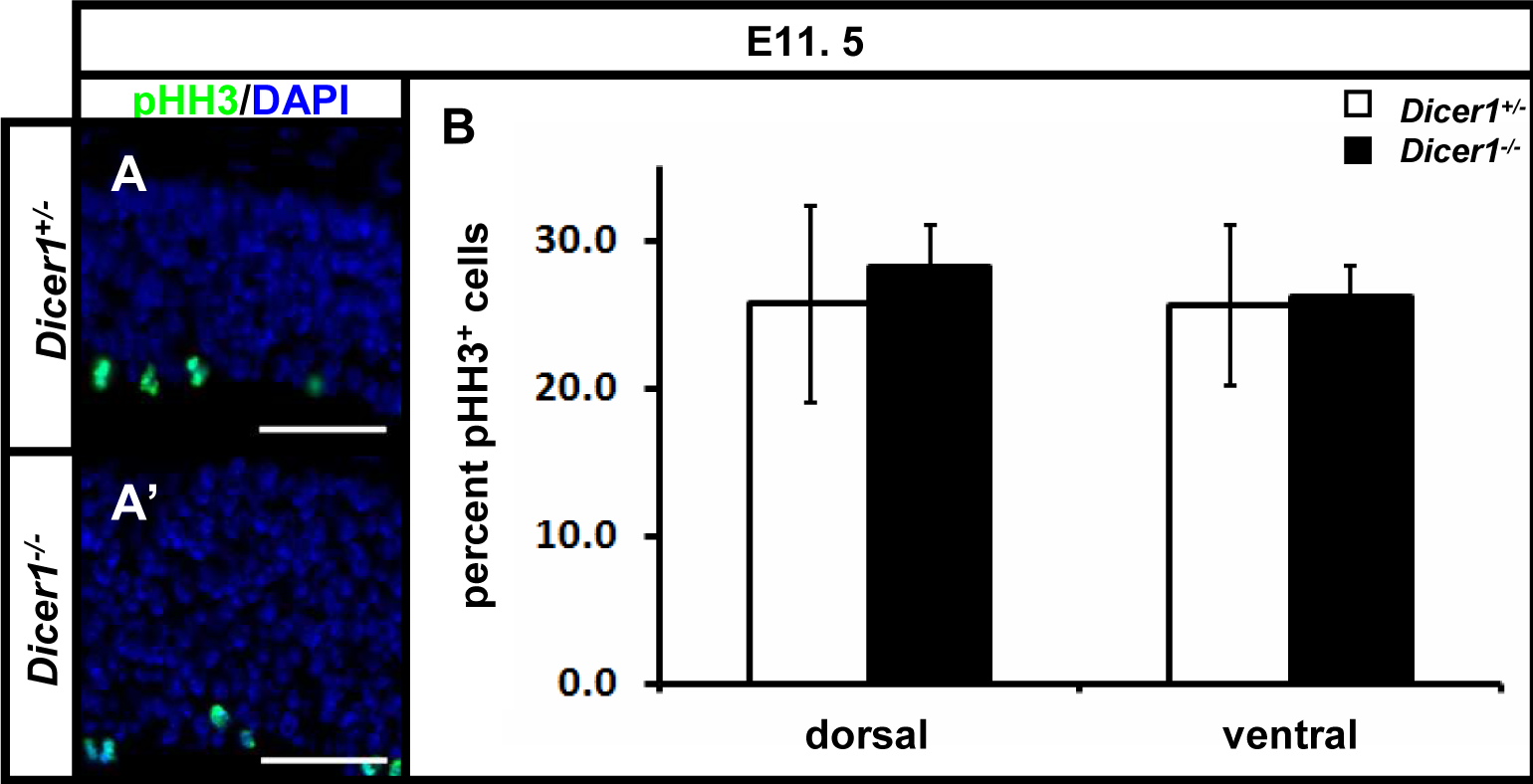

Supplement: Figure S1 — Proportion of mitotic cells remains unaltered after the loss of functional Dicer. Immunohistochemical staining using an antibody against phosphorylated (ser10) Histone 3 (pHH3) reveals that at E11.5 most immunoreactive cells are located directly at the ventricular surface (A) and loss of functional Dicer did not cause this pattern to be disrupted (A'). Quantification did not reveal any changes in the proportion of mitotic cells in either dorsal or ventral telencephalon (B). Scale bar: 50 µm, error bars indicate s.e.m. (TIF) [file pone.0023013.s001.tif]

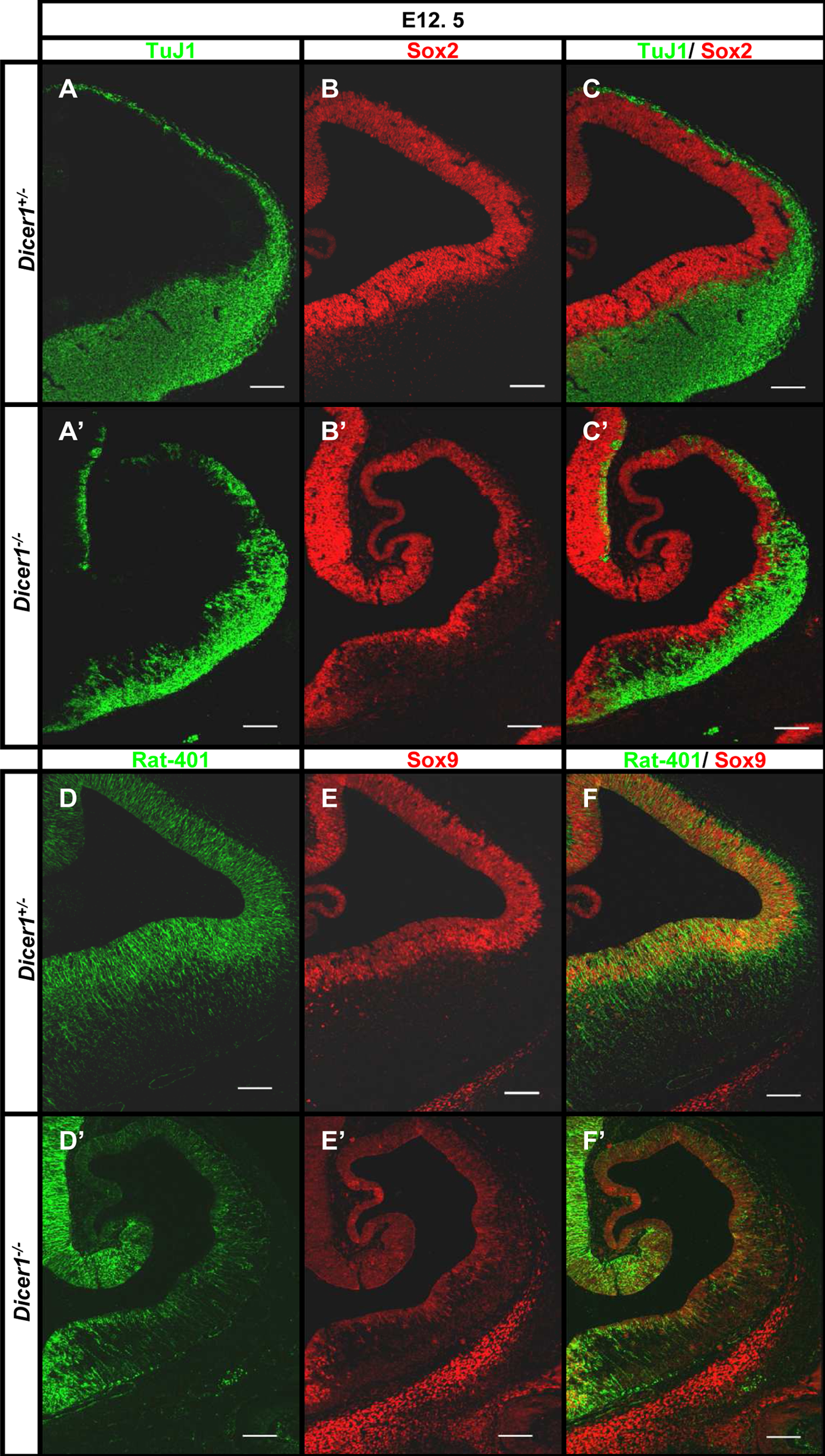

Supplement: Figure S2 — Dicer deficient telencephalon is severely disrupted by E12.5. Immunohistochamical staining for TuJ1 marks the postmitotic neurons in the telencephalon (A, C). This population is greatly reduced in Dicer1-/- telencephalon (A', C'). Sox2 marks the proliferative population (B, C), which is also diminished in the Dicer1-/- tissue (Figure S2 B', C'). At E12.5, radial glia express Rat-401 (D, F) as well as Sox9 (E, F) and in the Dicer1-/- telencephalon only a small fraction of radial processes can be detected (D', F') and the expression of Sox9 is greatly reduced (E', F'). Scale bar: 100 µm. (TIF) [file pone.0023013.s002.tif]

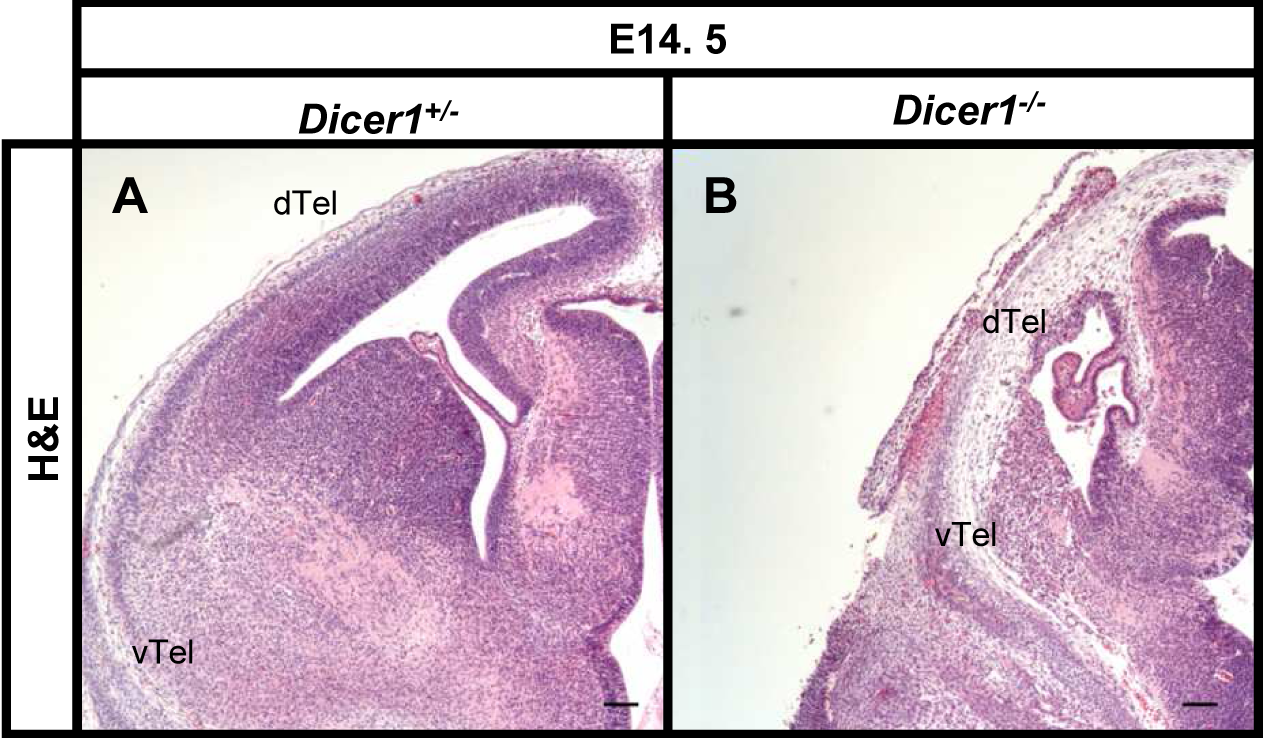

Supplement: Figure S3 — The volume of the Dicer1-/- telencephalic tissue is greatly reduced by E14.5. Hematoxylin and eosin staining of coronal sections through the brain at E14.5 (A) reveals a hugely abnormal telencephalon in the Dicer1-/- telencephalon (B). Scale bar: 100 µm. (TIF) [file pone.0023013.s003.tif]
